# Effect of hydrophilic polymers on the wettability, static and dynamic, of solid substrate covered by confluent monolayer of air-damaged SIRC cells

**DOI:** 10.1080/13102818.2014.997541

**Published:** 2015-01-13

**Authors:** Petar Eftimov, Nadezhda Stefanova, Zdravko Lalchev, Georgi As. Georgiev

**Affiliations:** ^a^ Department of Biochemistry, Faculty of Biology, Sofia University ‘St. Kliment Ohridski’, Sofia, Bulgaria; ^b^ Department of Cytology, Histology and Embriology, Faculty of Biology, Sofia University ‘St. Kliment Ohridski’, Sofia, Bulgaria

**Keywords:** dry eye syndrome, contact angle measurement, hydrophilic polymers

## Abstract

The aim of this study was to evaluate the possible implementation of hydrophilic polymers as recovery agents in air-damaged corneal cells. The sessile bubble technique was implemented to measure the wetting properties of four selected polymers: hydroxyethyl cellulose (HEC), sodium chondroitin sulphate (SCS), hydroxypropyl-methylcellulose (HPMC) and poloxamer F127 (PO12), at equilibrium conditions and in the case of advancing and receding contact angle. For testing the wetting properties of the polymers, glass slides covered with a confluent monolayer of Statens Seruminstitut rabbit cornea (SIRC) cells were used. HEC showed best properties for a broad concentration range, as the polymer showed capability to maintain low values of the static (equilibrium) contact angle (average static contact angle – 36.07˚, compared to average static compact angles of HPMC – 38.44˚, PO12 – 38.92˚ and SCS – 37.85˚), i.e. better wettability. Sessile bubble technique provides quick, relatively simple and reliable approach for testing surface properties of the listed polymers. The nature of the surface damage produced by the exposition of SIRC cells was used as a plausible model of evaporative dry eye syndrome, and thus the results may have clinical implementation.

## Abbreviations list

ANOVA–: analysis of variance
HEC–: hydroxyethyl cellulose
HPMC–: hydroxypropyl-methylcellulose
SCS–: sodium chondroitin sulphate
SIRC–: Statens Seruminstitut rabbit cornea
PO12–: poloxamer F127

## Introduction

According to the issues of 2007 International Dry Eye Workshop, ‘Dry eye is a multifactorial disease of the tears and ocular surface that results in symptoms of discomfort, visual disturbance and tear film instability with potential damage to the ocular surface. It is accompanied by increased osmolarity of the tear film and inflammation of the ocular surface’.[1] As it is an arising problem with prevalence of more than 60% in certain groups,[2] a lot of efforts are implemented by physicians and pharmaceutical manufacturers in search of ocular preparations, which may ameliorate that syndrome and related decrease in work productivity.[3]

Viscous polymer additives can improve the distribution and adhesion of the drop by interacting with the eye's mucosal layer. There is, however, an upper limit to patients’ tolerance to viscous eye drops, as they can cause blurred vision, reflex blinking and resistance to the eyelid's movements.[4] For the purpose of this study we chose four hydrophilic polymers: hydroxyethyl cellulose (HEC), sodium chondroitin sulphate (SCS), hydroxypropyl-methylcellulose (HPMC) and poloxamer F127 (PO12), which are compounds in commercial ocular preparations and tested their effect on the wettability of adherent Statens Seruminstitut rabbit cornea (SIRC) cell line. The choice of this rabbit cell line was based on the significantly lesser spontaneous blinking movements of this species (1/10 minutes [5]), compared to human (22.4 ± 8.9 per minute [6]). On the other hand, the inter-blinking time in the visual display users is prolonged, which is one of the predisposing factors of dry eye syndrome.[2] Simulated damage by a dry, warm airflow [7] over a solid surface covered with Statens Seruminstitut rabbit cornea cells (SIRC) cells and subsequent treatment with hydrophilic polymers, served as a plausible model system for quick and reliable evaluation of their wetting properties.

## Materials and methods

### Polymers

Selected polymers (Table 1) were tested within the pharmaceutically relevant scale, with an upper limit of the scale equal to the maximum applicable concentration allowed. The polymer concentrations were prepared via saline solution perfusion. Initially, the maximum pharmaceutically applicable concentration was applied in the solution, then a controlled volume of solution was washed out and thus, via dilution, a new polymer concentration was obtained. Once 100% saline solution perfusion was achieved, the measurement with the given polymer was completed.

**Table 1.** Main characteristics of polymers.

Name	CAS no.	Average *M*_w_	Concentrations used (%)	Manufacturer
HEC	71888-87-4	1,000,000	0.01, 0.10, 0.20, 0.40, 0.60	Sigma–Aldrich
HPMC	9004-65-3	75,000	0.01, 0.10, 0.20, 0.30, 0.40	Sigma–Aldrich
SCS	9082-07-9	500	0.01, 0.05, 0.10, 0.30, 0.50	Plantsman ltd.
PO12	9003-11-6	12,688	0.01, 0.025, 0.05, 0.075, 0.10	BASF

### Cell culture

SIRC cells (Sigma–Aldrich Chemie GmbH, Germany) were cultured on a standard no. 1.5 cover slip glass following a routine protocol and using a recommended culture medium (Eagle's minimum essential medium with added fetal bovine serum to a final concentration of 10%). The cells were cultured to 100% confluence and, prior to treatment with polymers, were exposed to warm dry air for three to five minutes.

#### Sessile bubble technique

The wetting properties of the polymers were measured via sessile bubble technique,[8,9] i.e. by measuring the contact angles between an axisymmetric air bubble submerged in 0.15 mol/L saline solution, pure or with dissolved polymer, put in contact with air-damaged SIRC cells. Three types of contact angles were measured.

1. Equilibrium contact angle (Figure 1) between the air bubble and the SIRC covered solid support. The air bubble is left static over the surface and is allowed to reach equilibrium with the solution and with the cell covered support. The equilibrium contact angle provides information about the overall wettability.

**Figure 1.**
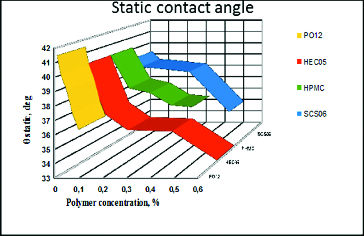
Dependence of the equilibrium contact angle (θ) between static air bubble and solid surface covered with air-damaged SIRC on polymer concentration. Zero per cent polymer concentration corresponds to control, i.e. pure saline solution with no polymer dissolved.
2. Advancing contact angle (Figure 2) between the bubble at contraction and the cell covered surface of the solid support. Advancing angle provides information on the wetting property of the polymers solution. Low advancing angle corresponds to better wetting abilities of the given polymer.

**Figure 2.**
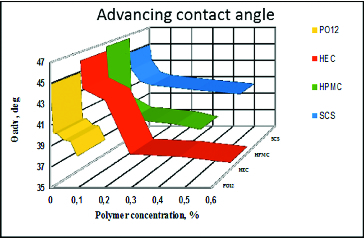
Dependence of the advancing contact angle (θ) between air bubble at contraction and solid surface covered with air-damaged SIRC on polymer concentration. Zero per cent polymer concentration corresponds to control, i.e. pure saline solution with no polymer dissolved.
3. Receding contact angle (Figure 3) between the bubble at expansion and the cell covered surface of the solid support. Receding angle provides information on the dewetting endurance of the polymer solution, i.e. lower values correspond to higher resistance to dewetting and stronger polymer–cell surface interaction.

**Figure 3.**
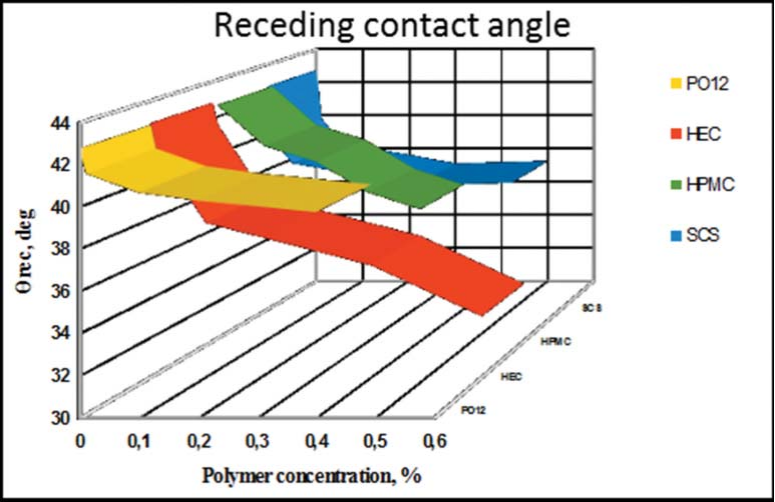
Dependence of the receding contact angle between air bubble at expansion and solid surface covered with air-damaged SIRC on polymer concentration. Zero per cent polymer concentration corresponds to control, i.e. pure saline solution with no polymer dissolved.

All contact angles measurements were repeated in triplicate and were performed on a CAM101 contact angle tension meter (KSV NIMA, Finland), with complete curve fitting based on the Young–Laplace equation.

### Statistical analysis

All the results were compared statistically with one-way analysis of variance (ANOVA) with GraphPad InStat software (GraphPad Software Inc., CA, USA) and all the values obtained at different polymer concentrations showed statistically significant difference, *P* < 0.05, with the control and between each other.

## Results and discussion

SIRC cells cultured on cover slips were used as a plausible model of corneal surface. In order to induce damage of the SIRC confluent layer, the cells were exposed to dry warm air for three to five minutes, until the equilibrium contact angle at the surface increased from 30°–33° to 42°–43°. It was possible to induce a stronger decrease in wettability with longer exposure of the cells, but we found that such loss of wettability was difficult to reproduce quantitatively and that it might result in cell detachment, loss of confluence and direct exposure of the solid support surface. For all the concentrations of a given polymer, we started by measurement of the static angle, followed by measurement of the advancing and the receding angle.

### Impact of the polymers on the equilibrium contact angle between static air bubble and solid surface covered with air-damaged SIRC cells

The data from the static angle measurements revealed a significantly better performance of HEC over HPMC, PO12 and SCS. HPMC maintained a low contact angle (better wettability) in pharmaceutically accepted concentrations. HEC showed best properties for a broad concentration range, as can be seen by the polymer capability to maintain low values of the static contact angle (Figure 1). It was susceptible to maximal dilution as compared with HPMC and SCS, but performed significantly better in all other concentrations. PO12 has a narrower therapeutic range and SCS maintained relatively good wettability only at the highest possible concentration. All the results are summarized in Table 2.

**Table 2.** Dependence of the average contact angle on the polymers concentration.

Polymer	HEC	HPMC	SCS	PO12
Concentration (%)	Average contact angle (°)			
(Control) 0.000	40.79	40.19	40.19	41.63
0.010	40.20 ± 0.08	38.46 ± 0.07	39.23 ± 0.09	41.49 ± 0.06
0.025	–	–	–	40.19 ± 0.08
0.050	–	–	38.56 ± 0.08	39.10 ± 0.08
0.075	–	–	–	37.46 ± 0.07
0.100	36.49 ± 0.04	38.39 ± 0.07	38.51 ± 0.04	36.37 ± 0.07
0.200	35.33 ± 0.05	38.04 ± 0.13	–	–
0.300	–	36.32 ± 0.12	38.17 ± 0.06	–
0.400	35.23 ± 0.16	35.51 ± 0.05	–	–
0.500	–	–	34.83 ± 0.15	–
0.600	33.11 ± 0.22	–	–	–

### Impact of the polymers on the dynamic (advancing and receding) contact angle between air bubble at contraction and solid surface covered with air-damaged SIRC cells

The results from the dynamic (advancing and receding) contact angles are presented in two formats.

**Figure 4.**
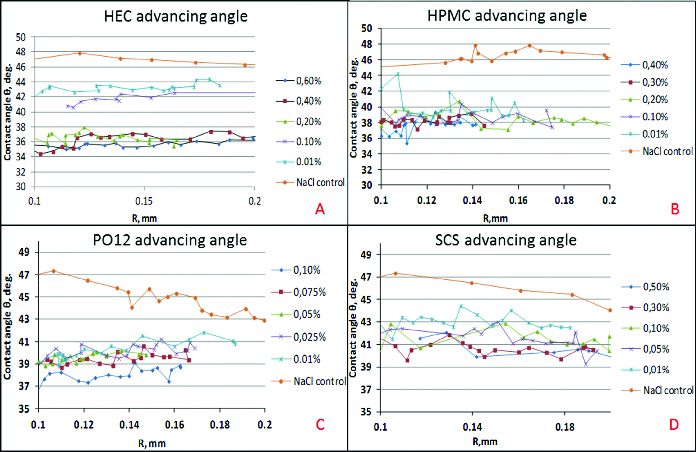
Dependence of the advancing contact angle (θ) on the distance travelled by the bubble edge (*R*) over the cell covered surface of the solid support for each polymer concentration: HEC (A), HPMC (B), PO12 (C), SCS (D).

**Figure 5.**
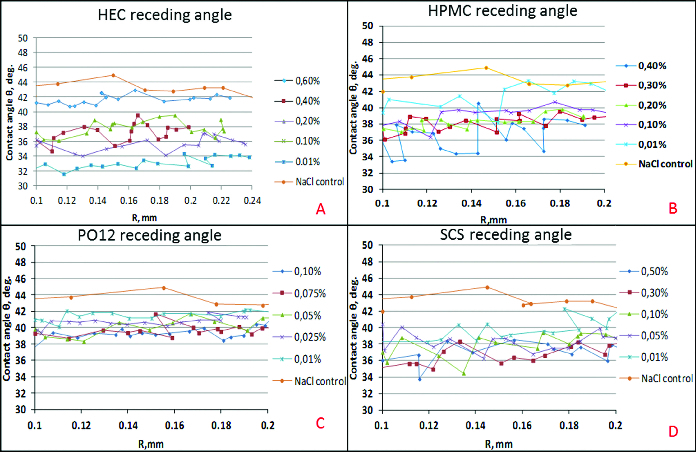
Dependence of the receding contact angle (θ) on the distance travelled by the bubble edge (*R*) over the cell covered surface of the solid support for each polymer concentration: HEC (A), HPMC (B), PO12 (C), SCS (D).

1. The dependence of the mean advancing/receding contact angle on polymer concentration for all the tested polymers. This format allows to compare between polymers (Figures 2 and 3).
2. The dependence of the mean advancing/receding contact angle on the distance travelled by the bubble edge over the cell covered surface of the solid support for each polymer concentration. This allows to evaluate how the surface heterogeneity, in terms of wettability, is smoothened by the polymer solution (Figures 4 and 5).

For the whole length of the surface at all concentrations, HEC improved the wettability of the surface both at advancing and receding conditions. HPMC was also able to recover the wettability of the cell covered surface over the entire tested surface, but it was quantitatively less efficient compared to HEC. HPMC showed major declinations in surface coverage in all the concentrations used. Moreover, in comparison with HEC, HPMC showed higher surface heterogeneity in terms of wettability of the treated SIRC-coated surfaces. The PO12 molecule performed with relatively homogenous coverage of the tested surface but was quantitatively less efficient compared to HEC and showed high susceptibility to dilution. SCS provided more uniform coverage of the surface, in comparison to HPMC and PO12, but proved to be of limited efficiency in terms of wettability, except in the maximal pharmaceutically acceptable concentration.

This study was performed without an animal model of dry eye, which is in conformity with the ethical guidelines of the Union of European Veterinary Practitioners. The study, however, has some limitations related with two main issues. First, the nature of the surface damage produced by the exposure of SIRC cells to air cannot be completely clarified and, second, the SIRC line is not of epithelial, but rather of fibroblastic origin (keratinocytes).[10] Still, the results may have clinical implementation but further studies would also be needed, e.g. with a human corneal epithelium cell line, which has been confirmed to express membrane associated mucins and after treatment of the cells with mucin-shedding agents.

## Conclusions

Sessile bubble technique provides quick, relatively simple and reliable approach for testing the surface properties of the polymers. This study was performed without an animal model of dry eye, which is in conformity with the ethical guidelines of the Union of European Veterinary Practitioners. The nature of the surface damage produced by the exposure of SIRC cells was used as acceptable model of evaporative dry eye syndrome, and thus the results may have clinical implementation. Therefore, it will be of value to perform identical experiments with a human corneal epithelium cell line, confirmed to express membrane associated mucins and after treatment of the cells with mucin-shedding agents.
